# Effects of eicosapentaenoic acid and docosahexaenoic acid on C2C12 cell adipogenesis and inhibition of myotube formation

**DOI:** 10.1080/19768354.2019.1661282

**Published:** 2019-09-03

**Authors:** Saeed Ghnaimawi, Sarah Shelby, Jamie Baum, Yan Huang

**Affiliations:** a Department of Animal Science, Division of Agriculture, University of Arkansas, Fayetteville AR, USA; b Department of Food Science, Division of Agriculture, University of Arkansas, Fayetteville AR, USA

**Keywords:** n-3 PUFAs, adipogenesis, myoblasts, EPA, DHA

## Abstract

Eicosapentaenoic acid (EPA) and docosahexaenoic acid (DHA) modulate cellular metabolic functions and gene expression. This study investigated the impacts of EPA and DHA on gene expression and morphological changes during adipogenic inducement in C2C12 myoblasts. Cells were cultured and treated with differentiation medium with and without 50 μM EPA and DHA. Cells treated with fatty acids had noticeable lipid droplets, but no formation of myotubes compared to control group cells. The expression levels of key genes relevant to adipogenesis and inflammation were significantly higher (*P *< 0.05) in cells treated with fatty acids. Genes associated with myogenesis and mitochondrial biosynthesis and function had lower (*P* < 0.05) expression with fatty acids supplementation. Moreover, fatty acid treatment reduced (*P* < 0.05) oxygen consumption rate in the differentiated cells. This suggested blocking myotube formation through supplementation with EPA and DHA drove myoblasts to enter the quiescent state and enabled adipogenic trans-differentiation of the myoblasts. Data also suggested that overdosage of EPA and DHA during gestation may drive fetal mesenchymal stem cell differentiation to the fate of adipogenesis and have a long-term effect on childhood obesity.

## Introduction

Growing bodies of evidence indicate that an increase in consumption of n-3 polyunsaturated fatty acids (PUFAs) is strongly linked to a decrease in risk factors for a variety of diseases (Seo et al. 2005). As well, n-3 PUFAs have shown to play promising roles in many biological processes including cognitive, learning, and visual development; the immune-inflammatory response; pregnancy outcomes; neurologic degeneration prevention; cardiovascular disease; and cancer intervention (Seo et al. 2005). Eicosapentaenoic acid (EPA, 20:5, n-3) and docosahexaenoic acid (DHA, 20:6, n-3) are two such n-3 PUFAs used for their beneficial effects on growth and development (Greenberg et al. 2008; Swanson et al. 2012; Janovska et al. 2013).

A recent series of trials found sheep fed a high energy diet to induce maternal obesity negatively affected the development of the muscular system in the fetus and offspring (Huang et al. 2010a; Huang et al. 2010b; Huang et al. 2012) whereas intramuscular fat tissue was elevated in the fetus at mid- and late-gestation (Zhu et al. 2006; Zhu et al. 2008). Due to its metabolic importance, fetal skeletal muscle is considered one of the target organs of lipid accumulation induced by a high energy maternal diet (Ong and Muhlhausler 2011; Kabaran and Besler 2015; Hsueh et al. 2018). Proper nutrient uptake and storage is essential for maintaining skeletal muscle mass and integrity. Coordination of muscle protein synthesis and proteolysis during normal physiological processes involves the cross-communication of multiple signal pathways. Imbalance of pathway networking can induce pathological conditions such as muscle mass atrophy, muscle aging, cancer, heart disease, and obesity (Le et al. 2014; Wagatsuma et al. 2016; Lipina and Hundal 2017).

EPA and DHA supplementation can modulate the expression of key regulatory genes related to myogenesis, adipogenesis, mitochondrial, and peroxisomal biosynthesis in murine C2C12 myoblasts during their normal differentiation into myocytes (Hsueh et al. 2018). Inhibiting myotube formation, upregulating adipogenic-related gene expression, promoting peroxisomal biogenesis, and suppressing mitochondrial activity were the primary outcomes of fatty acids treatment (FA) leading to decreased energy expenditure and ROS production to block myogenesis. This indicated that overdosage of n-3 PUFAs in the maternal diet influences fetal muscle development along with long-term effects on the development of metabolic diseases such as obesity and diabetes in adult offspring. However, the role of EPA and DHA in driving commitment of C2C12 cells into adipocytes lineage is not yet clear. Additionally, the role of concurrent treatment with EPA and DHA in determining the identity of adipocytes is lacking. Limited studies have addressed the molecular mechanisms behind their effect on regulating the metabolic function of brown and white adipocytes. Understanding the role of EPA and DHA in the commitment of C2C12 cells into a specific adipocyte phenotype would allow a better understanding of the development of obesity in an early life stage.

This study investigated the effect of combined EPA and DHA treatment on the induction of C2C12 myoblast cells into white adipocytes to explain the unusual accumulation of intramuscular fat depots and related insulin resistance involved in many metabolic disorders. *In vitro* models were used to mimic *in vivo* processes and isolate the desired experimental parameters. The hypothesis was that treatment with EPA and DHA would alter key pathways related to adipogenesis and mitochondrial biosynthesis in the differentiation of C2C12 cells.

## Materials and methods:

### Cell culture and differentiation

C2C12 cell culture was conducted as described previously (Klemm et al. 2001). Cells were then switched to differentiation media 2, containing only insulin (1 µg/ml) dissolved in ethanol for the next four days (Kim and Chen 2004; Yada et al. 2006; Yamanouchi et al. 2007). 50 μM EPA and 50 μM DHA were added to induction media 1 and 2 in the FA group.

### Oil Red O staining

Cells were then stained with filtered Oil Red O working solution and followed the method reported previously. (Konieczny and Emerson Jr 1984). Staining images were photographically produced using a Nikon DS-Fi3 digital camera mounted on a Nikon Eclipse TS 2R light microscope. 100% isopropanol was used to reduce background signal (Cheung et al. 2015).

### Real-time PCR

Total RNA was isolated from cells with TRIzol reagent. The cDNA was obtained using 5 Χ iScript cDNA synthesis kit (Bio-Rad, Richmond, CA) following manufacturer protocol. Real-time PCR was carried out using CFX Connect Real-Time PCR Detection System (Bio-Rad, Richmond, CA). The oligonucleotide primers (Table 1) used were designed with NCBI database and Primer Quest (IDT. com). Relative expression levels were normalized to 18s gene and expressed as fold change (Huang et al. 2010a).

**Table 1.** Primer sequences for real-time PCR.

Primers	Forward sequence	Reverse sequence
MyoD	TCTGGAGCCCTCCTGGCACC	CGGGAAGGGGGAGAGTGGGG
Myf-5	CCTGTCTGGTCCCGAAAGAAC	GACGTGATCCGATCCACAATG
Pax7	CTCAGTGAGTTCGATTAGCCG	AGACGGTTCCCTTTGTCGC
aP2	CGACAGGAAGGTGAAGAGCATCATA	CATAAACTCTTGTGGAAGTCACGCCT
C/EBPα	GCAAGCCAGGACTAGGAGAT	AATACTAGTACTGCCGGGCC
PPARγ	GATGTCTCACAATGCCATCAG	TCAGCAGACTCTGGGTTCAG
BMP-4	GCCCTGCAGTCCTTCGCTGG	CTGACGTGCTGGCCCTGGTG
TFAM	GCTTGGAAAACCAAAAAGAC	CCCAAGACTTCATTTCATT
PGC1α	TCCTCTGACCCCAGAGTCAC	CTTGGTTGGCTTTATGAGGAGG
COX7a1	CAGCGTCATGGTCAGTCTGT	AGAAAACCGTGTGGCAGAGA
mtDNA	CGATAAACCCCGCTCTACCT	AGCCCATTTCTTCCCATTTC
nDNA	CCTTGGGTCCTTGGCTTCGTTCCT	CTCAGCAATCAGCCGTCCAATTCCTA
PEX2	TGAAGGAACCACTTAGAAATTACAGA	CAGGGCCTTATTCAGTTCA
PEX19	CAGAGTGAGATGTGTTAGGAGATG	GTGCCAAGGAGACGAAGAC
PMP70	AAGAATGGCGATGGCAAGACT	TGTGAAACGGTAAAGAGGGTGAT
Il-6	GCTGGTGACAACCACGGCCT	AGCCTCCGACTTGTGAAGTGGT
TNFα	TGGGACAGTGACCTGGACTGT	TTCGGAAAGCCCATTTGAGT
18S	GTAACCCGTTGAACCCCATT	CCATCCAATCGGTAGTAGCG

### Oxygen consumption rate (OCR)

Orion Star A213 Dissolved Oxygen Meter (Thermo Scientific, Waltham, MA, USA) was used to measure the OCR (Zou et al. 2018).

### Western blot assay

Cells were scraped from the wells using PBS (1 ml/well) and then treated by lysis buffer (T-PER). Samples were separated on Mini-PROTEAN precast gels (Bio-Rad), then transferred onto Trans-Blot® Turbo™ Mini PVDF Transfer Packs (Bio-Rad). Immuno-staining was conducted with primary antibodies GAPDH (1:1000, Cusobio), myogenic differentiation 1 (MyoD) (1:25000, Cusobio), MyoG (1:25000, Cusobio), and PGC1α (1:1000, Abcam). The bands were visualized using ECL immunoblotting clarity system (Bio-Rad) and detected on ChemiDoc^TM^ Touch imaging system (Bio-Rad). Band density was normalized according to the Glyceraldehyde-3-phosphate dehydrogenase (GAPDH) content.

### Statistical analysis

All data from assays used to compare CON and FA groups were assessed for significance by the unpaired Student’s t-test with the assumption of equal variances, and arithmetic means ± SEM are reported. *P < *0.05 was considered statistically significant.

## Results

### EPA and DHA supplementation augmented trans-differentiation of C2C12 into white adipocyte-like phenotype through up-regulation of key adipogenic markers

**Figure 1.**
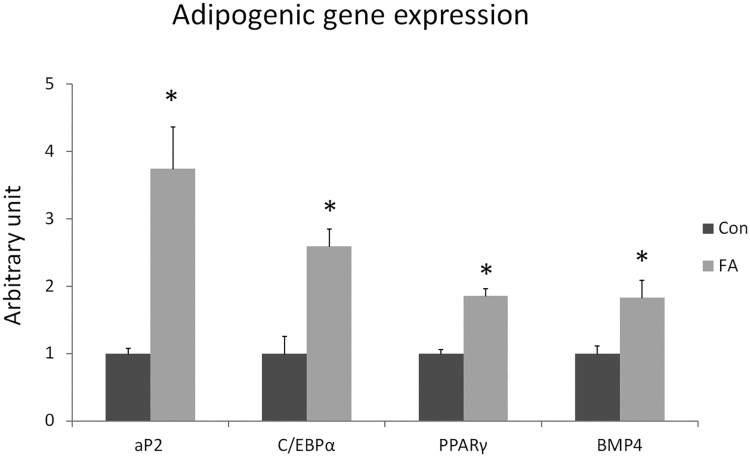
Gene expression analysis by RT-qPCR of adipogenic genes in C2C12 10 days after induction of white adipogenesis by a differentiation induction medium (DIM) in the absence (CON) or presence of 50 µM EPA and 50 µM DHA (FA). EPA and DHA were chronically present in the DIM (10 days). Data are expressed as mean + SEM. The relative expressions were calculated in arbitrary units. *P < 0.05; *n* = 6.

Peroxisome proliferator-activated receptor gamma (PPARγ), adipocyte protein 2 (aP2), CCAAT/enhancer binding protein-alpha (C/EBPα), and bone morphogenetic protein 4 (BMP4) are the most well-known master regulators of white adipogenesis. These genes were tested for up-regulation in C2C12 cells subjected to EPA and DHA treatments. The FA group exhibited a significant increase in basal levels of mRNA of aP2, C/EBPα, PPARγ, and BMP4 (274 ± 62.3%, *P* = 0.0017; 159 ± 25.3%, *P* = 0.0009; 85.2 ± 10.6%, *P* = 0.00002; and 82.9 ± 25.9%, *P = *0.0073, respectively). The CON group did not display an increase in the expression level of these genes even upon treatment with media containing adipogenesis-inducing agents (Figure 1).

### Concomitant treatment with EPA and DHA reduced mitochondrial function and biosynthesis

**Figure 2.**
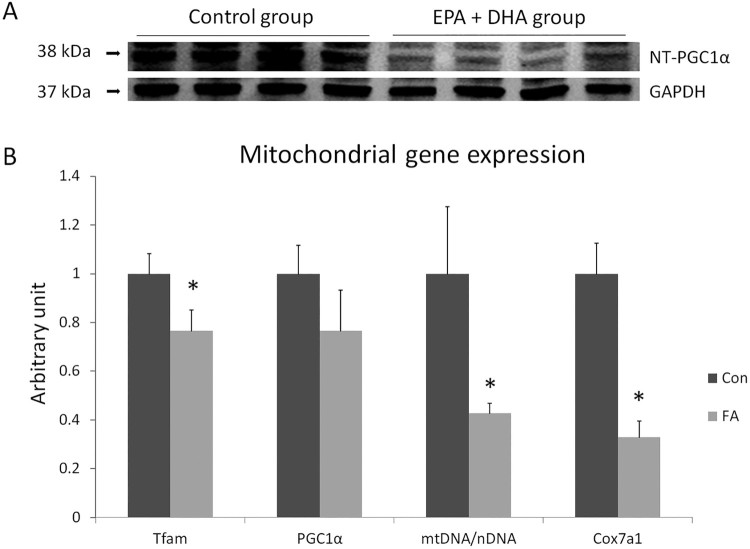
A- Protein level measurement by western blot of the spliced form of NT-PGC1a (a splice variant of PGC-1α, amino acids 1–270) and GAPDH from C2C12 cell culture 10 days after induction of white adipogenesis in the absence (CON) or presence of 50 µM EPA and 50 µM DHA (FA). 10% precast 10-well polyacrylamide gel (Bio-Rad) was used, *n* = 4. B- Gene expression analysis by RT-qPCR of gene-regulating mitochondrial function and biosynthesis in C2C12 10 days after induction of white adipogenesis by a differentiation induction medium (DIM) in the absence (CON) or presence of 50 µM EPA and 50 µM DHA (FA). EPA and DHA were chronically present in the DIM (10 days). Data are expressed as mean + SEM. Relative expressions were calculated in arbitrary units. *P < 0.05; *n* = 6.

The effect of EPA and DHA on mitochondrial function and biosynthesis was investigated by studying the expression of transcription factor A mitochondrial (TFAM), PPARγ co-activator 1 (PGC1α), cytochrome c oxidase subunit 7a1 (COX7a1), and the ratio of mitochondrial DNA (mtDNA) to nuclear DNA (nDNA) as markers essential to assess the thermogenic capacity of adipocytes. TFAM, mtDNA/nDNA, and COX7a1 were substantially down-regulated (23.3 ± 8.45%, *P* = 0.0389; 57.1 ± 3.98%, *P* = 0.0335; and 67.1 ± 6.74%, *P* = 0.0004, respectively), indicating that EPA and DHA supplementation inhibited mitochondrial function and biosynthesis (Figure 2).

### EPA and DHA inhibited multinucleated myotube formation

**Figure 3.**
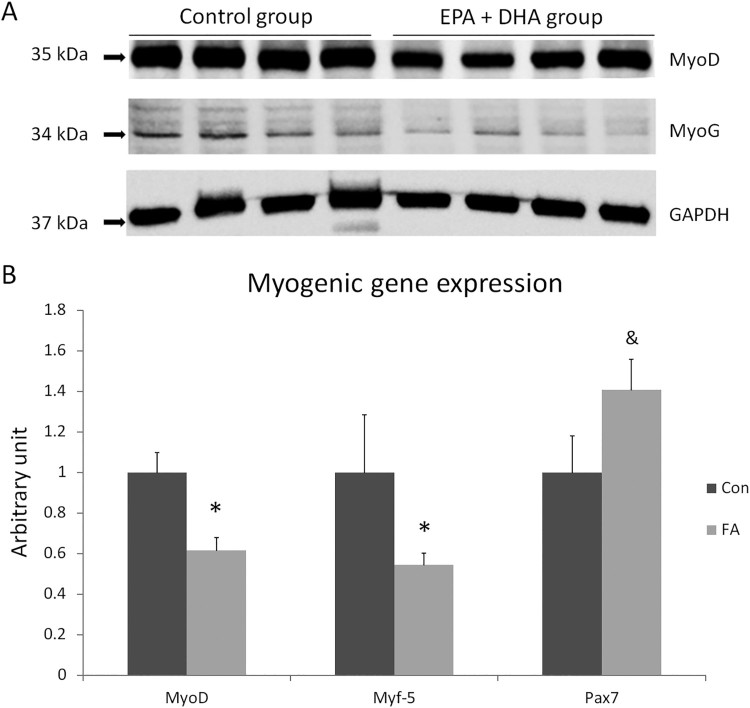
A- Protein level measurement by western blot of MyoD, MyoG, and GAPDH from C2C12 after induction of white adipogenesis in the absence (CON) or presence of 50 µM EPA and 50 µM DHA (FA). 10% precast 10-well polyacrylamide gel (Bio-Rad) was used, *n* = 4. B- Gene expression analysis by RT-qPCR of myogenesis regulating genes in C2C12 after induction of white adipogenesis by a differentiation induction medium (DIM) in the absence (CON) or presence of 50 µM EPA and 50 µM DHA (FA). EPA and DHA were chronically present in the DIM (10 days). Data are expressed as mean + SEM. The relative expressions were calculated in arbitrary units. * *P *< 0.05; & *P *< 0.1; *n* = 6.

MyoD and myogenic factor 5 (Myf5) genes were selectively down-regulated in the FA group in comparison with CON group (38.5 ± 6.49%, *P *= 0.0028; and 45.6 ± 5.79%, *P *= 0.037, respectively), while up-regulation of paired box 7 (Pax7) tended to be highly expressed in the FA group (40.7 ± 15.1%, *P* = 0.0569) (Figure 3).

### EPA and DHA promoted fat droplet accumulation

**Figure 4.**
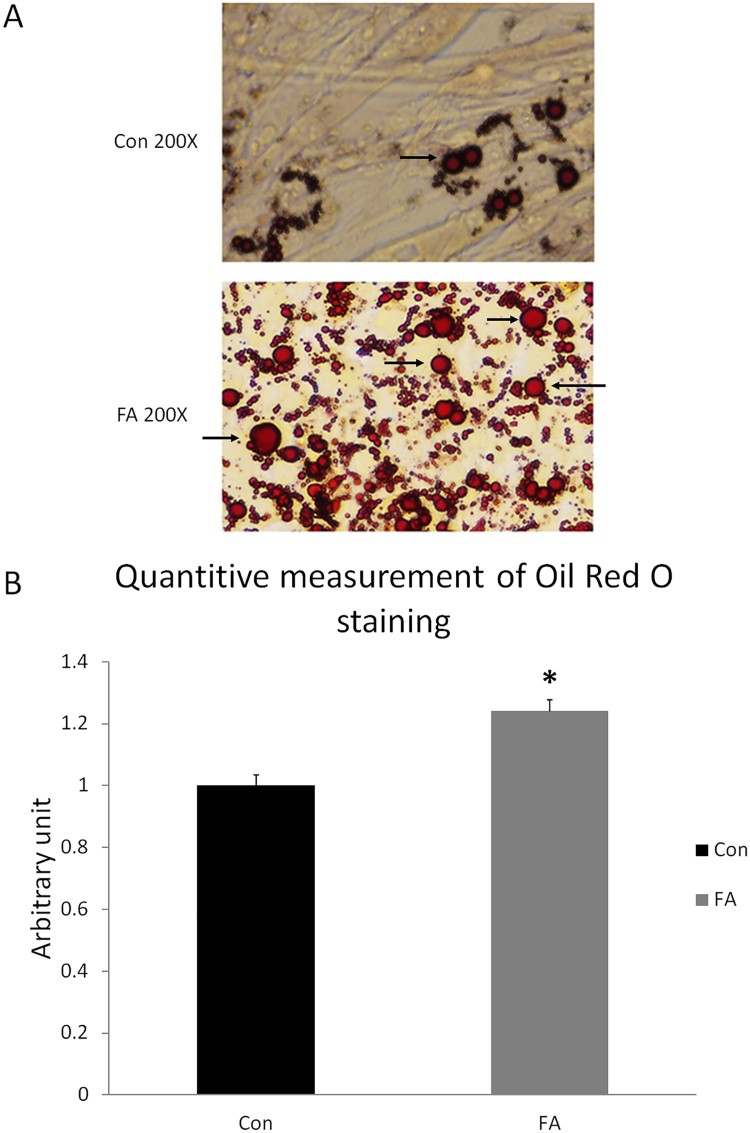
Representative images of Oil Red O staining of C2C12 myoblasts after adipogenic differentiation. (**A**) Treatment with EPA and DHA (FA) showed excessive accumulation of distributed lipid clusters. Lipid droplets are indicated with black arrows. (B) Quantitative assessment of Oil Red O staining in FA and CON. Signiﬁcant differences between the two groups are at the indicated time points. * *P < *0.05; *n* = 6. Data were normalized by the total number of cells counted using a hemocytometer in each group.

The FA group showed higher accumulation of lipid droplets compared with the CON group (Figure 4(A)). Quantitative measurement of Oil Red O staining showed a significant increase in the amount of stain extracted from the FA group compared to CON (24.2 ± 3.41%, *P = *0.0001) (Figure 4(B)). These results suggested that EPA and DHA induced the trans-differentiation of myoblasts into adipocytes.

### EPA and DHA affected the metabolism of C2C12 myoblasts

**Figure 5.**
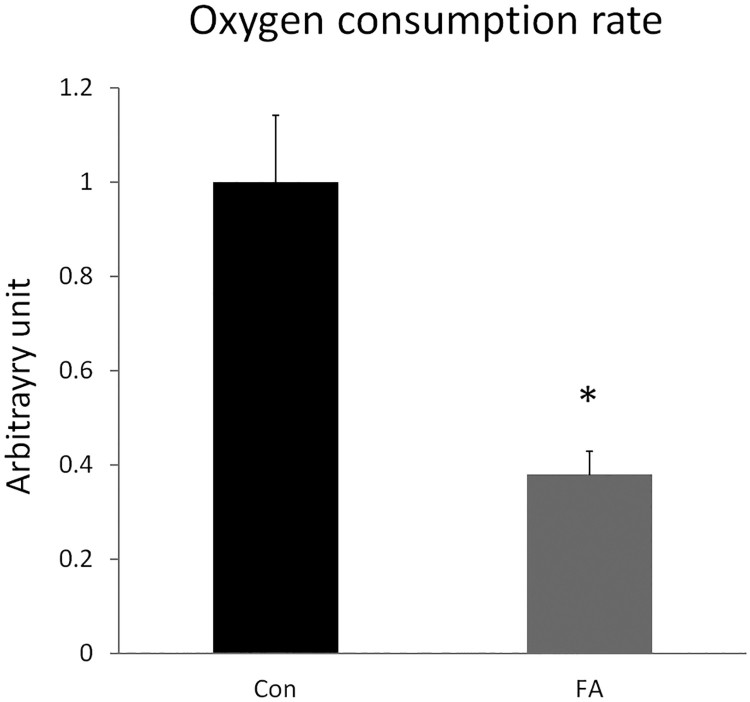
Relative oxygen consumption rate in C2C12 cells 10 days after induction of white adipogenesis by a differentiation induction medium (DIM) in the absence (CON) or presence of 50 µM EPA and 50 µM DHA (FA). EPA and DHA were chronically present in the DIM (10 days). Data are expressed as mean + SEM. The relative oxygen consumption rate was measured for 15 min and calculated in arbitrary units. * *P *< 0.05; *n* = 6.

The FA group showed a significant decrease in oxygen consumption rate when compared to the CON group (61.9 ± 4.95%, *P = *0.0016) (Figure 5). This decline is consistent with the decreased expression seen in mitochondrial biosynthesis related genes. The FA group showed an increase in the expression of genes driving lipogenesis and suppressing mitochondria induced thermogenesis, which may be responsible for the dramatic reduction in the maximal respiratory capacity. The data indicated that EPA and DHA supplementation negatively affected mitochondrial activity.

### Morphological changes in C2C12 myoblasts

**Figure 6.**
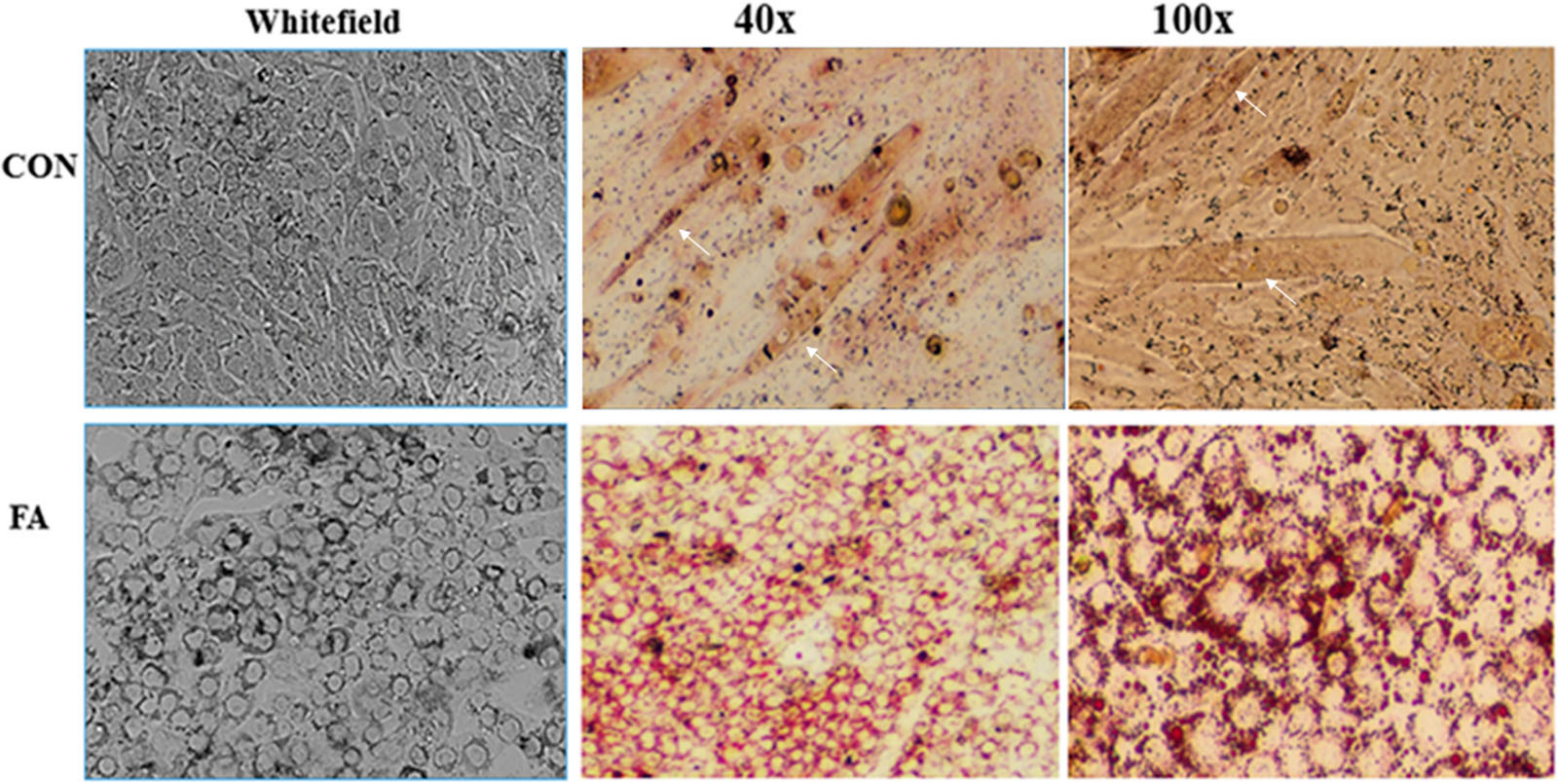
Morphological changes of C2C12 myoblasts under EPA and DHA treatment (FA). Arrows refer to multinucleated tubes in the control group (CON). The CON group displays long, flattened, aligned striation pattern typical in the morphology of myotubes (white arrows). The FA group displays the large, round lipid droplet characteristic of white adipocytes with large amounts of cytoplasm and a single nucleus.

It was previously shown that inhibition of myotube formation would induce myocyte trans-differentiation into other lineages (Singh 2007). In this experiment, morphological changes during myoblast differentiation were detected by Oil Red O staining (Figure 6). RT–PCR and Oil Red O staining results confirmed trans-differentiation of C2C12 cells into adipocyte, indicated by the absence of multinucleated myotubes and the presence of clusters of rounded cells containing lipid droplets in the FA group compared to the CON group.

### Pro-inflammatory effect of EPA and DHA treatment

**Figure 7.**
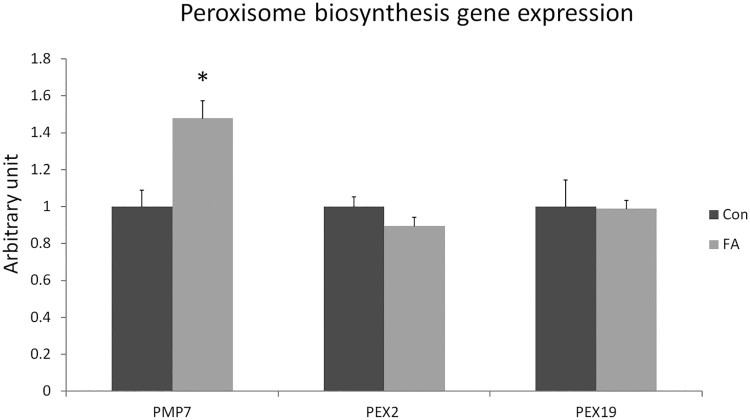
Gene expression analysis by RT-qPCR of gene-regulating cytokines production in C2C12 10 days after induction of white adipogenesis by a differentiation induction medium (DIM) in the absence (CON) or presence of 50 µM EPA and 50 µM DHA (FA). EPA and DHA were chronically present in the DIM (10 days). Data are expressed as mean + SEM. The relative expressions were calculated in arbitrary units. * *P *< 0.05; *n* = 6.

A study suggested that inﬂammation in adipose tissue boosts systemic inﬂammation, which is highly related to insulin resistance in obesity (Ajuwon and Spurlock 2005). The potential capacity of EPA and DHA to modulate inﬂammation in C2C12 cells trans-differentiated into adipocytes were tested. EPA and DHA treated group exhibited a dramatic increase in interleukin 6 (IL-6) gene expression (74.0 ± 28.6%, *P = *0.0214), but not tumor necrosis factor α (TNFα) (*P* = 0.3080) (Figure 7).

### Effect of EPA and DHA treatment on genes involved in peroxisome biogenesis and function

**Figure 8.**
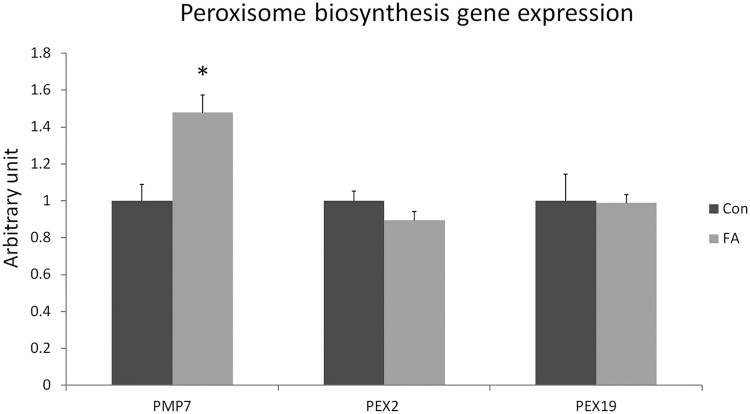
Gene expression analysis by RT-qPCR of gene-regulating peroxisomes function and biogenesis in C2C12 cells 10 days after induction of white adipogenesis by a differentiation induction medium (DIM) in the absence (CON) or presence of 50 µM EPA and 50 µM DHA (FA). EPA and DHA were chronically present in the DIM (10 days). Data expressed as mean + SEM. The relative expressions were calculated in arbitrary units. * *P *< 0.05; *n* = 6.

ATP binding cassette subfamily D member 3 (70 kDa peroxisomal membrane protein, PMP70) was significantly up-regulated (47.8 ± 9.64%, *P = *0.0018) in the FA group (Figure 8). However, peroxisomal biogenesis factor 2 (PEX2) and PEX19, specifically associated with the biogenesis process, were not significantly affected (*P* = 0.0728 and *P *= 0.4698).

## Discussion

Long-chain PUFAs play an essential role in the fetus and neonatal development (Jensen 1999; Duttaroy 2009; Kabaran and Besler 2015). Studies indicate insufficient maternal n-3 PUFAs supplement results in greater childhood body and abdominal fat mass of the offspring (Vidakovic et al. 2016). However, few studies have focused on the effects of excessive n-3 fatty acids on growth and development (Church et al. 2008; Hsueh et al. 2018). In previous research with n-3 PUFAs supplementation in C2C12 cell cultures, toxicity of EPA and DHA on C2C12 cells was found with concentrations above 50 μM in the growth medium. The myogenic feature of C2C12 cells was inhibited while adipogenic potential was promoted by 50 μM EPA and DHA treatment (Lee et al. 2013; Hsueh et al. 2018).

Previous research also found trans-differentiation of C2C12 myoblasts accompanied changing myoblast morphology. Excessive accumulation of lipid droplets in the cytoplasm was induced by thiazolidinediones, a potent adipogenic agent (Singh 2007). Regarding arrested formation of multinucleated myotubes and adipocyte creation, our data suggested that adipocyte morphology and blunting the formation of multinucleated myotubes could be attributed to increased expression of C/EBPα and PPAR-γ and decreased expression of myosin heavy chain.

Consistent with a previous study using a chick model (Shang et al. 2014), increased expression of adipogenesis-related genes, including PPARγ and aP2, was associated with cytoplasmic lipid droplet accumulation and adipocyte differentiation induced by fatty acid treatment. Further, increased expression of the master regulator of adipocyte differentiation, PPARγ, and adipocyte-specific genes C/EBPα and aP2 in FA group enhanced cytoplasmic lipid droplet accumulation and changed cell morphology from fibroblast-like into polygon (Matsubara et al. 2008). This demonstrated that trans-differentiation of cell lines altered adipogenic potential through up-regulation of adipogenic transcription factors PPARγ and C/EBPα.

Transcription factor Pax7 is a myogenesis related gene that plays a vital role in guiding the lineage specification of myocytes. Pax7 is highly expressed during the proliferation phase, but rapidly down-regulated during differentiation. It has been shown that inhibiting myotube formation in C2C12 cells is closely associated with up-regulation of Pax7 (Xia et al. 1996). A study demonstrated myoblast differentiation and myotube formation were induced only when the expression of Pax7 was inhibited by MyoD, mediated by the expression of miR-206 and -486, which target the Pax7 gene 3′-untranslated region (Dey et al. 2011). Factors inhibiting expression of these micro RNAs can block myotube formation through assisting Pax7 protein synthesis. Preventing myotube formation with thiazolidinediones could drive myoblast development into another lineage. Accordingly, this study suggested that EPA and DHA mediated increased expression of Pax7 may be implicated in C2C12 cells trans-differentiation into adipocytes. On the other hand, Pax7 up-regulation may be a compensatory attempt to counter adipogenicity and regenerate the impaired myogenic capacity of C2C12 cells.

Our data suggested that high fatty acid treatment down-regulated expression of genes involved in mitochondrial function and biosynthesis, including TFAM, COX7a1, and mtDNA/nDNA ratio. TFAM is a transcription factor critical for mitochondrial replication encoded in the nucleus (Fisher and Clayton 1988). It has been shown that mitochondrial function and subsequent cell growth and morphology can be compromised by TFAM gene knockdown, followed by reducing mtDNA copy number and expression (Jeng et al. 2008). Consistent with previous studies, impaired TFAM expression negatively affected the mtDNA/nDNA (mitochondrial DNA per nuclear DNA copy number) ratio and mitochondrial respiratory chain efficiency (Wang et al. 2001; Woo et al. 2012; Xie et al. 2016; Lan et al. 2017). Decreased mitochondrial biosynthesis was shown by the mtDNA/nDNA ratio (Phillips et al. 2014; Hsueh et al. 2018).

The transcription of mitochondrial genes regulating lipid metabolism is well-orchestrated by PPARγ, through regulation of PGC1 expression, which maintains the normal physiological and the functional status of mitochondria (Vega et al. 2000; Puigserver and Spiegelman 2003). Mitochondrial gene expression can also be regulated by the expression of TFAM. An example of the importance of TFAM and COX7a1 in promoting mitochondrial biosynthesis and maintaining its structure is evident in a study in which the eﬀects of linoleic acid on cell viability after streptozotocin damage occurred through maintaining the mitochondrial structure and increased biosynthesis induced by up-regulation of TFAM, COX7a1, and PGC1 (Jeng et al. 2009). Accordingly, our data suggested there was no defect in the PPARγ-PGC1 pathway. Reduction in mitochondrial biosynthesis, function, and, subsequently, decreased oxygen consumption rate was attributed to the disruption of PGC1-NRF-TFAM cascade.

To further test the pro-adipogenic effects of n-3 PUFAs on mitochondrial metabolism, we calculated metabolic rates by measuring the oxygen consumption rate. Our data suggested that EPA and DHA reduced the metabolic capacity of trans-differentiated C2C12 cells. In addition to the adverse effect of down-regulation of TFAM and COX7a1, the decreased oxygen consumption rate was linked to up-regulation of adipogenic markers. The oxygen consumption rates were consistent with those obtained from qPCR. This suggested a diminished oxygen consumption rate in the FA group was attributed to the enhanced expression PPARγ and its target gene aP2 along with the increased expression of C/EBPα. Correspondingly, it was reported that reducing expression of PPARγ and its target genes in liver and adipose tissue is accompanied by increased AMP/ATP ratio (den Besten et al. 2015). Therefore, stimulating oxidative metabolism through activation of AMP-activated protein kinase (AMPK) pathway plays an essential role in shifting lipid synthesis to fatty acids oxidation to reduce adipogenesis in mice. Moreover, induced activation of AMPK was blocked by adding rosiglitazone, the PPARγ agonist (McGarry et al. 1983; den Besten et al. 2015). The induction of adipogenic markers, including PPARγ, ap2, and C/EBPα, are strongly linked with suppression fatty acid oxidation, stimulation of lipogenesis, and triglyceride deposition (den Besten et al. 2015).

BMP4, a white adipogenesis specific marker, was highly expressed in FA group as compared CON group, which may account for the acquisition of white adipocytes-like phenotype and impairment of the thermogenic capacity. Other studies have demonstrated that BMP4 can promote the shift of adipocytes from brown to white during the terminal differentiation phase due to suppression of lipolysis via regulation of hormone-sensitive lipase expression. BMP4 stimulated the expression of adipogenic markers such as PPARγ and ap2, leading to enhanced lipid storage. Further, BMP4 blunted not only cAMP-induced lipolysis, but also lipolysis stimulated by isoproterenol, forskolin, and isobutylmethylxanthine and suppressed the acute rise in the OCR promoted by cAMP (Modica et al. 2016).

The FA group exhibited a significant increase in the expression of the pro-inflammatory cytokine IL-6 but not TNFα. Our findings consistent with results that reported freshly isolated human adipocytes could secrete profound amounts of IL-6 in the supernatants aspirated from the culture media. It has been shown that IL-6, secreted mainly from adipose tissue in humans, contributes 15–35% of the total circulating level cytokines (Fried et al. 1998). TNFα, on the other hand, is scantly produced by human fat cells (Fried et al. 1998; Ekin et al. 2003).

As a transcriptional factor, C/EBPα, plays a pivotal role in regulation and expression of IL-6 promoter (Vanden Berghe et al. 2000). Consistently, increased expression of C/EBPα gene in this study was implicated in promoting IL-6 expression. Because adipocytes are a significant source of pro-inﬂammatory cytokines, and a promotor of IL-6 receptor and IL-6 expression in obese patients (Maachi et al. 2004; Sindhu et al. 2015), it was expected that TNFα expression increase as well. However, it was demonstrated that IL-6 has the potential to inversely affect the expression and production of TNFα in skeletal muscle (Pedersen et al. 2003).

Adipocytes are infiltrated with a considerable number of peroxisomes, appearing as small organelles adjacently localized to lipid droplets in adipocytes(Schrader et al. 2013). Their numbers are dramatically increased during adipocyte differentiation, reflecting their involvement in lipid metabolism (Novikoff and Novikoff 1982). Various aspects of peroxisomal biogenesis, including their assembly, division, and inheritance, are orchestrated by specific proteins called peroxins, which are encoded by PEX genes, such as PEX2 and PEX19 (Dimitrov et al. 2013; Lodhi and Semenkovich 2014). It was reported that n-3 PUFAs promote peroxisome synthesis bio-activity and results in increased FA beta-oxidation (Willumsen et al. 1993; Neschen et al. 2002; Hirabara et al. 2013; Romanatto et al. 2014). Moreover, PMP70 is unnecessary for biogenesis and proliferation but required for the normal function of peroxisomes (Imanaka et al. 1999). Consistent with these results, this study showed that with increased expression of PMP70 in EPA and DHA treated group (47%) but not PEX2 or PEX19, PUFAs affected peroxisomal function, exhibited by increased fatty acid transport into peroxisome lumens, without affecting the biogenesis process. Data suggested up-regulation of PMP70 as a compensatory mechanism prompted by EPA and DHA associated lipid droplet accumulation.

## Conclusions

This study suggested that concomitant treatment with EPA and DHA inhibits myotube formation by targeting myogenesis signature genes such as Myf5, MyoD, MyoG, and Pax7, while also promoting the white-like phenotype, as indicated by up-regulation of white adipose-selective genes such as BMP4, C/EBPα, and IL-6. This study also indicated that combined supplementation of EPA and DHA is correlated with mitochondrial dysfunction, attributed to a reduction in expression of TFAM, COX7a1, and PGC1α genes regulating mitochondrial biosynthesis and function. Lipid droplet accumulation was associated with reduced oxygen consumption rate. EPA and DHA supplementation were able to further promote lipid storage over induction differentiation media alone, attributed to the higher number of adipocytes formed. Finally, this study demonstrated that white adipocyte-like subsets could arise from mesenchymal precursors expressing Myf5, previously thought to give rise only to skeletal muscle and brown adipocytes. These observations suggested that excessive maternal exposure to EPA and DHA may potentially be involved in obesity and negatively affect muscle tissue development, mass, and quality in offspring.
